# Recovery after Taking a Large Dose of Hydrocyanic Acid

**Published:** 1854-04

**Authors:** W. M. Burman

**Affiliations:** Wath-upon-Dearne


### SELECTIONS.

From the London Lancet.

### *Recovery after taking a large dose of Hydrocyanic Acid.* By W. M. Burman, Esq., M.R.C.S.E., Wath-upon-Dearne.

The occurrence I am about to relate took place on August 6th, 1853, at six P. M.; but before going into the detail I must premise that my father and myself are practising together, and use the same surgery. We keep our Scheele's hydrocyanic acid for dispensing much diluted,—namely, in the proportion of one minim of the acid to a fluid drachm of water; this is kept in four-ounce purple glass bottle. It so happened that a short time previously we had received a fresh supply of Scheele's acid in a bottle precisely similar to the one in which we keep our *very dilute* acid. This strong acid was put into a cupboard under lock and key, but on the day in question had been taken out to replenish the dispensing bottle, and then left on the surgery counter. Soon afterwards my father returned from his afternoon ride, and being troubled with slight dyspepsia, went into the surgery, as was his habit, and mixed himself a draught containing a little aromatic spirit of ammonia and bicarbonate of soda in two ounces of water, adding to it a fluid drachm of the *very dilute* acid, *as he thought*, but he took it out of the bottle of Scheele's acid standing on the counter. He drank this off, noticing nothing peculiar in the taste. In a few seconds afterwards, upon looking at the bottle, he thought the label appeared cleaner than usual, and the idea flashed on his mind that perhaps he had taken the strong acid. A glance at the proper dispensing bottle, which was in its place, confirmed this. He directly poured half an ounce of aromatic spirit of ammonia into the measure, and drank it, *he believes* without water, but he may have added some. He then walked into the house to the bottom of the stairs (a distance of about sixteen yards), and called me; I came down stairs directly and went into the surgery, where I found him standing at the counter with a glass measure in his hand. He said. "I have taken a drachm of the acid; what can be done?" adding, "I have since taken half an ounce of aromatic spirit of ammonia." There was nothing in his appearence to attract my attention, but he spoke hurriedly, expiring deeply at the same time; he then sat down on a chair. I immediately put some crystals of sulphate of iron into a measure, adding one drachm of the tincture of the sesquichloride of iron, and one ounce of water, stirring the mixture vigorously, to get as many of the crystals dissolved as possible in the time, and gave it to him to drink, which he did. I had no liquor potassium at hand, or I should have added some to the mixture before giving it to him, to precipitate the mixed oxides of iron; as it was, I was obliged to trust to the ammonia he had taken. My object in giving him this was to endeavour to get the mixed oxides of iron in contact with a least part of the acid in the stomach, and thus to convert it into Prussian blue, as suggested by Messrs. Smith of Edinburgh. Directly after drinking this, he began to breathe deeply, pressed his hand over his forehead and top of his head, and said: "Oh, I feel very queer; had I not better go out of doors?" I said "Yes" and with my assistance he staggered out, and dropped senseless in a sitting posture on a stone step at the surgery door. This was just about *two minutes* after taking the poisin, as ascertained afterwards by going through the same routine. From this time he recollects nothing till twenty minutes afterwards. His breathing was now slow and very deep; his eyes turned up completely under the upper lid; pulse moderate in volume, but very slow and intermitting. I hastily took off his neckerchief, fetched a large pitcher of water, and poured it over the back of his head and down his back. This produced no apparent effect at the instant, but a few seconds afterwards he vomited a mouthful of glairy mucus. This was nearly four minutes from the time of taking the poison. I sent for another pitcher of water, and during the interval again examined the pulse, and found it slower than before, feeble, and threatening to stop altogether; respiration very slow and deep, irregular, with blowing expiration and puffing of the cheeks; a little frothy mucus ran from his mouth. At this period the relaxation of the whole body was as great as that of a person recently dead. I now gave him another dose of the iron mixture as before, adding a little aromatic spirit of ammonia. By speaking loudly, shaking him, and pouring it into his mouth, I got him to swallow the whole of it.

Meantime the servant returned with another pitcher of water (about four pints), which I instantly poured down bis back (five minutes after taking the poison). This appeared to rouse him a little, and directly afterwards he vomited about two ounces of a deep bluish-green liquid; this I was glad to see, for I hoped that partial decomposition of the acid had taken place. The pulse and respiration were slightly quicker and more regular; pupils still quite invisible; conjunctiva injected; face livid. After an inspiration more than usually deep, he opened his mouth wide, and stretched out his arms; a spasmodic flutter passed over the face, and then over all the body, and I expected fully to hear a scream, as I had generally noticed in animals, or that he was about to have a convulsion ; but just as it begin to pass off, he vomited twice, and seemed relieved by it. The vomited matter was still bluish-green, and this time mixed with pieces of half-digested meat, which had been taken at dinner, four hours previously. I next gave him two drachms of aromatic spirit of ammonia in one ounce of water, and with some difficulty got him to swallow it. Having again filled the pitcher I poured the water down his back as before, and directly afterwards he vomited two or three times (seven minutes from the time of taking poison). The respiration and pulse were now very much better, the latter being quicker and pretty regular. Soon after the vomiting, I noticed a little movement of the hands, apparently voluntary. I now hoped the immediate danger was passed (eight minutes), so I watched the symptoms for a minute or two, and then the pulse beginning to falter a little. I gave him two drachms of aromatic spirit of ammonia in one ounce of water, which, with a little difficulty, he swallowed. I tried to get him to speak, or to give any evidence that he understood what was said to him, but failed to elicit any sign of consciousness from him ; still I considered him tolerably safe, for the pulse was pretty strong and regular ; the countenance was also now regaining its natural appearance, and the extreme flaccidity of the limbs was nearly gone. After about five minntes more had elapsed (fifteen minutes), he opened his eyes, and gave utterance to an ejaculation, and evidently understood, to some extent, what was said to him. Soon afterwards he complained of his trousers being wet, and this is the first circumstance that he remembers after leaving the surgery more than twenty minutes before. We presently got him into the house, and gave him a little hot brandy-and-water. I was now anxious to get him to bed as quickly as possible, judging that rest in the recumbent position would most conduce to his recovery. After sitting ten or fifteen minutes, he was able with great difficulty and with our assistance to walk up-stairs, but vomited several times during the transit. We got him into bed, and soon afterwards, turning to me, he said, "You should have used the cold affusion," and seemed quite surprised, when I told him that I had done so pretty freely. During the evening, he took a simple effervescent draught now and then, and presently he dropped asleep. He passed a good night, and next morning complained of pain across the loins, but was otherwise pretty well, except a feeling of general weakness. I ought to have mentioned that my father is about sixty years of age, and of a strong constitution.

A day or two afterwards I analysed the acid. I measured one drachm in the same two-ounce measure that my father used, and put it into a minim measure ; it filled up to seventy minims. I diluted this and then added a solution of nitrate of silver till no more precipitate was produced. I repeatedly washed this precipitate, and dried it carefully for a long time; but to make sure that it contained no more water. I put it into a warm oven for an hour, during which time it lost nearly one-fifth of a grain It now weighed exactly twelve grains, which would be equal to 2.4 grains of anhydrous hydrocyanic acid, *the quantity my father took*. As 100 grains of the acid filled up to 105 minims in the same small measure, this would give 3.3 as the per centage of the anhydrous acid in the Scheele's acid used.

I have been thus minute, perhaps unnecessarily so, in the detail of this case, because it presents many points of unusual interest; some of these it may be well to recapitulate very briefly: —

1stly. This is the largest quantity recorded (so far as I know) after taking which recovery has ensued.

2ndly. The quantity of acid taken was *measured*, being certainly no less than one drachm of Scheele's strength (at 3.3 per cent.), and being equal to 2.4 grains of real acid.

3rdly. The time at which insensibility supervened after taking the poison is accurately known, namely *two minutes*.

Lastly. The good effects of the cold affusion and the probable decomposition of part of the poison by the mixed oxides of iron.

